# Impact of the COVID-19 pandemic on antidepressant prescribing with a focus on people with learning disability and autism: an interrupted time series analysis in England using OpenSAFELY-TPP

**DOI:** 10.1136/bmjment-2024-301378

**Published:** 2025-04-28

**Authors:** Christine Cunningham, Orla Macdonald, Andrea Schaffer, Andrew Brown, Milan Wiedemann, Rose Higgins, Chris Bates, John Parry, Louis Fisher, Helen Curtis, Amir Mehrkar, Liam C Hart, William Hulme, Victoria Speed, Tom Ward, Richard Croker, Christopher Wood, Alex Walker, Colm Andrews, Ben Butler-Cole, David Evans, Peter Inglesby, Iain Dillingham, Simon Davy, Lucy Bridges, Thomas O’Dwyer, Steve Maude, Rebecca Smith, Amelia Green, Ben Goldacre, Brian MacKenna, Sebastian Bacon

**Affiliations:** 1 Nuffield Department of Primary Care Health Sciences, University of Oxford, Oxford, UK; 2 Oxford Health NHS Foundation Trust, Oxford, UK; 3 University of Oxford, Oxford, UK; 4 Nuffield Department of Primary Care Health Sciences, The Bennett Institute for Applied Data Science, University of Oxford, Oxford, UK; 5 TPP, Leeds, UK; 6 The Phoenix Partnership, Leeds, UK

**Keywords:** COVID-19, PSYCHIATRY, Depression

## Abstract

**Background:**

COVID-19 restrictions led to increased reports of depressive symptoms in the general population and impacted health and social care services. We explored whether these changes affected antidepressant prescribing trends in the general population and those with learning disability or autism.

**Methods:**

With the approval of NHS England, we used >24 million patients’ primary care data from the OpenSAFELY-TPP platform. We used interrupted time series analysis to quantify trends in those prescribed and newly prescribed an antidepressant across key demographic and clinical subgroups, comparing pre-COVID-19 (January 2018–February 2020), COVID-19 restrictions (March 2020–February 2021) and recovery (March 2021–December 2022) periods.

**Results:**

Prior to COVID-19 restrictions, antidepressant prescribing was increasing in the general population and in those with learning disability or autism. We did not find evidence that the pandemic was associated with a change in antidepressant prescribing trend in the general population (relative risk (RR) 1.00 (95% CI 0.97 to 1.02)), in those with autism (RR 0.99 (95% CI 0.97 to 1.01)) or in those with learning disability (RR 0.98 (95% CI 0.96 to 1.00)).

New prescribing post restrictions was 13% and 12% below expected had COVID-19 not happened in both the general population and those with autism (RR 0.87 (95% CI 0.83 to 0.93), RR 0.88 (95% CI 0.83 to 0.92)), but not learning disability (RR 0.96 (95% CI 0.87 to 1.05)).

**Conclusions and implications:**

In this England study, we did not see an impact of COVID-19 on overall antidepressant prescribing, although unique trends were noted, such as trends in new antidepressant prescriptions which increased in care homes over the pandemic and decreased in the general population and those with autism since recovery.

**WHAT IS ALREADY KNOWN ON THIS TOPIC**

- The prescribing of antidepressants in the UK has been increasing for more than a decade.
- Studies globally have found differing impacts of COVID-19 on mental health outcomes in the general population, by age, sex, socioeconomic status and care home status.

**WHAT THIS STUDY ADDS**

- This study gives a detailed account of antidepressant prescribing trends in England during the COVID pandemic and recovery period through to December 2022.
- It addresses important questions about how healthcare services responded to the mental health burden during this time, especially in those with learning disability or autism.

**HOW THIS STUDY MIGHT AFFECT RESEARCH, PRACTICE OR POLICY**

- This study demonstrates how the pandemic did not lead to an increase in antidepressant prescriptions in the general population, but more studies are needed to ensure that antidepressants are used appropriately within vulnerable populations.
- Improvements are needed in the documentation of diagnosis when prescribing medicines.

## Background

 The prescribing of antidepressants in the UK has been increasing for more than a decade.^1^ During the COVID-19 pandemic, the Office for National Statistics Opinions and Lifestyle Survey found the proportion of individuals reporting depressive symptoms during COVID restrictions was close to double prepandemic levels.^2^ Yet, concerns have been raised about potential overprescribing of antidepressants, particularly to those with mild depression.^3^

In 2016, the Stopping Over Medication of People with a learning disability, autism or both (STOMP) initiative was introduced to reduce inappropriate prescribing or overprescribing in those groups.^4^ In 2019, NHS Digital (NHSD) introduced indicators to support STOMP, including tracking the proportion of patients with learning disability prescribed an antidepressant without an active depression diagnosis. Comparing 2016–2017 to 2020–2021, they recorded an increase from 10.8% to 11.6%.^5^ During the COVID-19 pandemic, some services offered to people with learning disability or autism and their families and carers were suspended or restricted,^6^ and literature from that period has described the negative impact the restrictions had on people with learning disability and autism, such as increases in anxiety and depressive symptoms.^7 8^

OpenSAFELY is a secure analytics platform for electronic patient records built by our group on behalf of NHS England to deliver urgent academic and operational research during the pandemic.^9^ We set out to use the OpenSAFELY platform to assess the impact of the COVID-19 pandemic on antidepressant prescribing in patients in the general population, those with learning disability and/or autism and in key demographic and clinical subgroups.

## Methods

### Study design

With the approval of NHS England, we performed an interrupted time series analysis (ITSA) to examine changes in monthly rates of registered patients prescribed an antidepressant for the 5-year period from January 2018 to December 2022. We compared the 26 months from January 2018 to February 2020 (pre-COVID-19), 12 months from March 2020 to February 2021 (restrictions) and 22 months from March 2021 to December 2022 (recovery).

The UK government announced its COVID response plan on 3 March 2020 and legal restrictions came into force on 26 March.^10^ Exposure to restrictions was defined as March 2020 through February 2021. The recovery period was defined from March 2021 as primary and secondary schools reopened on 8 March 2021, suggesting a return to some essential activities. The ITSA allowed us to use the respective populations as their own controls, taking into account pre-existing trends (the counterfactual) and seasonality in antidepressant prescribing, comparing these against the actual trends that occurred during and after the pandemic.

### Data source and processing

The dataset analysed within OpenSAFELY is based on >24 million people currently registered with General Practice (GP) surgeries using TPP SystmOne software. All data were linked, stored and analysed securely using the OpenSAFELY platform, https://www.opensafely.org/, as part of the NHS England OpenSAFELY COVID-19 service. Data include pseudonymised data such as coded diagnoses, medications and physiological parameters. No free text data are included. All codes are shared openly for review and reuse under Massachusetts Institute of Technology (MIT) open licence. Detailed pseudonymised patient data are potentially reidentifiable and therefore not shared. Data management and analysis was performed using Python V.3. Codes for data management and analysis, as well as codelists, are archived online at https://github.com/opensafely/antidepressant-prescribing-lda. As Enhancing the Quality and Transparency of Health Research guidelines for ITSA are still under development, the Jandoc *et al* recommendations were followed.^11^

### Study population

We included all alive individuals who were registered at an OpenSAFELY-TPP practice at the end of each month, across the study period. Those with unknown age and sex were excluded as their small numbers would have necessitated redactions of the next smallest group to avoid potential reidentification of individuals. Those excluded are described in online supplemental table S1.

### Study outcomes

Antidepressants were classified using British National Formulary codes. Some specific antidepressants were excluded as they were more commonly used for indications other than depression. Full codelists and details on the codelist development methodology can be found on www.opencodelists.org and are linked in online supplemental table S2.

Patients with more than one antidepressant prescription in a month were only counted once. This analysis reflects prescribing rather than dispensing or use. We count prescription issues. Prescription durations greater than 1 month and/or intermittent non-compliance resulting in prescription intervals to be longer than 1 month would result in a patient not being counted every single month.

### Population characteristics

We describe the population in October 2022 as it was close to the end of the study period and prescribing in November and December has been shown to be subject to more seasonal variation.^12^ We calculated the prescribing rates for all and individual types of antidepressant per 1000 patients. Counts of ≤5 were redacted, with remaining counts rounded to the nearest 10 to avoid potential reidentification.

Monoamine Oxidase Inhibitors (MAOI) and other antidepressants were combined into a single ‘other’ group. Those prescribed two different classes of antidepressants in the single month (eg, Selective Serotonin Reuptake Inhibitors (SSRI) and other) were counted as ‘multiple’.

Learning disability and autism diagnoses were defined using the NHSD primary care reference sets.^13^ Patients with both learning disability and autism were counted in both groups.

Additional variables were defined for age (0–19, 20–29, 30–39, 40–49, 50–59, 60–69, 70–79 and 80+ years), sex, 2019 Index of Multiple Deprivation (IMD) as quintiles, ethnicity according to the 2001 census, the nine English geographical regions and care home status. Ethnicity may be self-reported or entered by a clinician or administrator. The OpenSAFELY-TPP population has been shown to be broadly representative of the English population, but there is higher coverage in the East of England (91%) and lower coverage in London (19%).^14^ Care should be taken when interpreting regional rates, as they could reflect other demographic differences.

Diagnosis groups were defined as whether a patient was on the Quality and Outcomes Framework (QOF) depression register (active depression diagnosis), had an anxiety diagnosis, both or neither. QOF business rules for the depression register include logic to exclude resolved diagnoses.^13^ The anxiety codelist was developed based on a keyword search for ‘anxiety’ or ‘anxious’ within listed SNOMED CT (Systemized Nomenclature of Medicines - Clinical Terms) codes. Inclusion/exclusion details can be found on opencodelists (online supplemental table S2). By QOF definition, this measure is limited to those aged 18 and older.

Patient demographics were extracted monthly, except ethnicity, which for reduced computational time used the latest recorded code.

### Antidepressant prescribing during the COVID-19 pandemic

Our primary aim was to understand whether the interruptions caused by the COVID-19 restrictions impacted antidepressant prescribing overall and in at-risk learning disability and autism populations. Secondary aims were to assess the impact in other demographic or clinical subgroups.

#### Overall prescribing

Counts of patients with any antidepressant prescription per month were modelled with a Poisson regression. The log of the population was included as an offset term to compute a rate rather than a count, given that the registered population was increasing over time.

The model included variables representing the pre-COVID-19 trend (slope), a step and slope change for the COVID-19 restrictions and a step and slope change for the start of the recovery period. March 2020 and April 2020 were extreme outliers and coded as dummy variables. One pair of Fourier terms was included to adjust for seasonality. We explored non-linear trends with time, by including a squared term for month of follow-up, but it did not improve the model fit. To account for autocorrelation and heteroscedasticity, robust SEs were computed using the Newey-West method with a lag of 2 being applied.

ITSA was illustrated with scatter plots of the observed data, a line showing the model fitted data and a dotted line showing the rate assuming the pandemic did not happen (no COVID-19 counterfactual). The counterfactual was computed using the fitted model to estimate the rate with the step, slope and outlier variables set to 0.

We summarise the data in two ways. First, we report a rate difference between the model fitted value with the pandemic in the last month of the study period and its counterfactual without the pandemic. Second, we compute the ratio of the model fitted value with the pandemic over its counterfactual without the pandemic for each month (relative risk or RR). An overall estimate of whether postrestriction prescribing was below the no COVID-19 counterfactual was computed by taking the geometric mean of those ratios, and its CI, using a method proposed by Travis-Lumer *et al*.^15 16^

We defined ‘New prescribing’ as patients who had been started on an antidepressant in the study period and had not received an antidepressant prescription for the previous 2 years.

For the demographic and clinical subgroups, we created separate models for each subgroup (one model using only data for ages 0–19, another model using only data for ages 20–29, etc).

#### Sensitivity analysis

We repeated our population ITSA analysis using data from OpenPrescribing. OpenPrescribing is an openly available viewer of primary care prescription reimbursement data which contain information on prescription volumes at an aggregate practice level, but do not contain individual patient data such as age or indication.^17^ These data have been used by others to assess antidepressant prescribing.^1 18^

## Results

### Population characteristics

In January 2018, there were 23 864 380 registered patients, increasing to 25 504 380 in December 2022. In October 2022, 2 048 040 patients were prescribed an antidepressant, a prescribing rate of 80.5 per 1000 patients (table 1, online supplemental table S3). The rate of prescribing was higher in women than men (107.0 vs 53.9 per 1000). Prescribing increased with increasing IMD (least deprived 69.7 vs most deprived 93.2 per 1000) and increasing age (age group 0–19, 5.4 vs 80+, 139.2 per 1000) and was highest in white ethnicity (98.5 per 1000). Of patients aged 18+ receiving an antidepressant prescription, 30% had neither an active depression diagnosis nor a record of anxiety.

**Table 1:** Characteristics of registered patients in October 2022 by total population, learning disability or autism

		All		Learning disability		Autism	
		Prescribed antidepressant, n (%)	Registered patients, n (%)	Prescribed antidepressant, n (%)	Registered patients, n (%)	Prescribed antidepressant, n (%)	Registered patients, n (%)
Total		2 048 040 (100.0)	25 455 570 (100.0)	26 810 (100.0)	145 920 (100.0)	31 360 (100.0)	264 610 (100.0)
Age band	0–19	30 200 (1.5)	5 582 550 (21.9)	1060 (4.0)	29 370 (20.1)	5240 (16.7)	152 540 (57.7)
	20–29	185 450 (9.1)	3 130 490 (12.3)	5210 (19.4)	32 690 (22.4)	12 080 (38.5)	64 050 (24.2)
	30–39	273 300 (13.3)	3 671 930 (14.4)	5460 (20.4)	27 030 (18.5)	6360 (20.3)	25 340 (9.6)
	40–49	323 540 (15.8)	3 269 820 (12.8)	4270 (15.9)	17 660 (12.1)	3450 (11.0)	10 450 (3.9)
	50–59	431 840 (21.1)	3 458 490 (13.6)	5330 (19.9)	19 380 (13.3)	2770 (8.8)	7830 (3.0)
	60–69	355 050 (17.3)	2 816 370 (11.1)	3690 (13.8)	13 020 (8.9)	1130 (3.6)	3410 (1.3)
	70–79	269 310 (13.1)	2 237 650 (8.8)	1500 (5.6)	5510 (3.8)	280 (0.9)	830 (0.3)
	80+	179 350 (8.8)	1 288 270 (5.1)	300 (1.1)	1240 (0.8)	50 (0.2)	140 (<0.1)
Care home	False	1 982 340 (96.8)	25 280 560 (99.3)	19 670 (73.4)	122 820 (84.2)	28 930 (92.3)	257 480 (97.3)
	True	65 700 (3.2)	175 010 (0.7)	7140 (26.6)	23 100 (15.8)	2430 (7.7)	7120 (2.7)
Diagnosis	Anxiety	537 670 (26.4)	2 636 550 (12.9)	8020 (30.5)	22 260 (18.2)	9280 (32.3)	30 860 (23.6)
	Both	598 340 (29.4)	1 728 650 (8.5)	5280 (20.1)	10 310 (8.4)	10 200 (35.5)	24 550 (18.7)
	Depression register	287 140 (14.1)	1 076 850 (5.3)	3240 (12.3)	6660 (5.4)	2960 (10.3)	8580 (6.6)
	Neither	614 500 (30.2)	14 977 500 (73.3)	9780 (37.2)	83 390 (68.0)	6330 (22.0)	66 980 (51.1)
Ethnicity	Asian or Asian British	56 840 (2.8)	1 823 980 (7.2)	790 (2.9)	8880 (6.1)	440 (1.4)	8510 (3.2)
	Any other Asian background	11 560 (0.6)	428 410 (1.7)	130 (0.5)	1460 (1.0)	100 (0.3)	1840 (0.7)
	Bangladeshi	4770 (0.2)	127 600 (0.5)	50 (0.2)	780 (0.5)	30 (<0.1)	1030 (0.4)
	Indian	18 770 (0.9)	744 810 (2.9)	220 (0.8)	2250 (1.5)	160 (0.5)	2350 (0.9)
	Pakistani	21 740 (1.1)	523 170 (2.1)	380 (1.4)	4400 (3.0)	160 (0.5)	3290 (1.2)
	Black or black British	17 600 (0.9)	634 380 (2.5)	260 (1.0)	2900 (2.0)	210 (0.7)	4430 (1.7)
	African	7880 (0.4)	411 600 (1.6)	60 (0.2)	1320 (0.9)	60 (0.2)	2470 (0.9)
	Any other black background	3900 (0.2)	106 530 (0.4)	90 (0.3)	740 (0.5)	70 (0.2)	1060 (0.4)
	Caribbean	5830 (0.3)	116 250 (0.5)	110 (0.4)	850 (0.6)	80 (0.3)	910 (0.3)
	Mixed	15 870 (0.8)	387 750 (1.5)	240 (0.9)	2090 (1.4)	420 (1.3)	5650 (2.1)
	Any other mixed background	5550 (0.3)	144 210 (0.6)	90 (0.3)	760 (0.5)	160 (0.5)	1990 (0.8)
	White and Asian	3160 (0.2)	83 440 (0.3)	50 (0.2)	390 (0.3)	80 (0.3)	1070 (0.4)
	White and black African	2300 (0.1)	74 160 (0.3)	20 (<0.1)	310 (0.2)	50 (0.2)	860 (0.3)
	White and black Caribbean	4870 (0.2)	85 950 (0.3)	80 (0.3)	630 (0.4)	130 (0.4)	1720 (0.7)
	Other ethnic groups	15 250 (0.7)	538 860 (2.1)	130 (0.5)	1110 (0.8)	160 (0.5)	2240 (0.8)
	Any other ethnic group	13 320 (0.7)	343 990 (1.4)	100 (0.4)	900 (0.6)	120 (0.4)	1670 (0.6)
	Chinese	1930 (<0.1)	194 880 (0.8)	30 (0.1)	220 (0.2)	40 (0.1)	570 (0.2)
	White	1 660 980 (81.1)	16 863 830 (66.2)	22 680 (84.6)	109 240 (74.9)	24 230 (77.3)	177 810 (67.2)
	Any other white background	111 760 (5.5)	2 350 800 (9.2)	1120 (4.2)	6720 (4.6)	1310 (4.2)	12 070 (4.6)
	British	1 538 070 (75.1)	14 400 910 (56.6)	21 470 (80.1)	102 100 (70.0)	22 810 (72.7)	165 140 (62.4)
	Irish	11 150 (0.5)	112 130 (0.4)	100 (0.4)	420 (0.3)	110 (0.4)	600 (0.2)
	Missing	281 490 (13.7)	5 206 760 (20.5)	2700 (10.1)	21 680 (14.9)	5890 (18.8)	65 960 (24.9)
IMD	1—Most deprived	468 560 (22.9)	5 025 740 (19.7)	7960 (29.7)	42 570 (29.2)	7110 (22.7)	65 080 (24.6)
	2	412 720 (20.2)	4 912 660 (19.3)	6320 (23.6)	32 880 (22.5)	6720 (21.4)	55 600 (21.0)
	3	420 100 (20.5)	5 224 810 (20.5)	5470 (20.4)	28 830 (19.8)	6590 (21.0)	52 900 (20.0)
	4	372 570 (18.2)	4 909 320 (19.3)	3920 (14.6)	22 200 (15.2)	5450 (17.4)	44 700 (16.9)
	5—Least deprived	315 030 (15.4)	4 517 350 (17.7)	2560 (9.5)	16 040 (11.0)	4560 (14.5)	37 540 (14.2)
	Missing	59 050 (2.9)	865 690 (3.4)	580 (2.2)	3400 (2.3)	930 (3.0)	8770 (3.3)
Region	East	478 510 (23.4)	5 831 400 (22.9)	6080 (22.7)	30 420 (20.8)	6920 (22.1)	56 400 (21.3)
	East Midlands	391 670 (19.1)	4 427 330 (17.4)	4900 (18.3)	25 040 (17.2)	6380 (20.3)	52 560 (19.9)
	London	55 040 (2.7)	1 816 040 (7.1)	520 (1.9)	5710 (3.9)	620 (2.0)	9930 (3.8)
	North East	121 680 (5.9)	1 184 850 (4.7)	1620 (6.0)	7950 (5.4)	2050 (6.5)	16 860 (6.4)
	North West	215 360 (10.5)	2 203 770 (8.7)	2890 (10.8)	15 170 (10.4)	2980 (9.5)	24 660 (9.3)
	South East	118 880 (5.8)	1 654 200 (6.5)	1750 (6.5)	9500 (6.5)	2280 (7.3)	18 440 (7.0)
	South West	289 920 (14.2)	3 527 600 (13.9)	4070 (15.2)	21 320 (14.6)	5400 (17.2)	40 970 (15.5)
	West Midlands	72 470 (3.5)	1 031 020 (4.1)	1090 (4.1)	6600 (4.5)	1210 (3.9)	11 840 (4.5)
	Yorkshire and the Humber	297 980 (14.5)	3 691 560 (14.5)	3790 (14.1)	23 770 (16.3)	3420 (10.9)	32 160 (12.2)
	Missing	6520 (0.3)	87 800 (0.3)	90 (0.3)	440 (0.3)	110 (0.4)	770 (0.3)
Sex	F	1 360 440 (66.4)	12 709 460 (49.9)	13 130 (49.0)	57 170 (39.2)	12 850 (41.0)	72 380 (27.4)
	M	687 600 (33.6)	12 746 120 (50.1)	13 680 (51.0)	88 750 (60.8)	18 510 (59.0)	192 230 (72.6)

Patients can be in both the learning disability and autism subgroups.

Counts have been rounded to the nearest 10 and the rate computed with the rounded numbers.

Group sums may not exactly match the ‘“Total’” due to rounding.

IMD, Index of Multiple Deprivation.

In October 2022, there were 145 920 patients with recorded learning disability and 264 610 with recorded autism. Rates of antidepressant prescribing were higher among those with learning disability (183.7 per 1000) than autism (118.5 per 1000). Rates per 1000 of prescribing without a diagnosis of anxiety or depression were 117.3 in learning disability and 94.5 in patients with autism, compared with 41.0 in the general population, although if we consider those who received a prescription, 37% with learning disability had neither an active depression diagnosis nor a record of anxiety, compared with 22% for those with autism and 30% in the general population. Demographic prescribing trends remained similar to the total population, except for IMD where the correlation was less strong.

The most prescribed antidepressant class in October 2022 was SSRIs (54%), followed by tricyclics (18%) and ‘other’ (20%) (online supplemental table S4). Nine per cent of patients were prescribed more than one class. There was a greater proportion of SSRIs prescribed (68%, 70%) and relatively fewer tricyclics (7%, 5%) for those with learning disability or autism.

### Trends in overall prescribing

**Figure 1:**
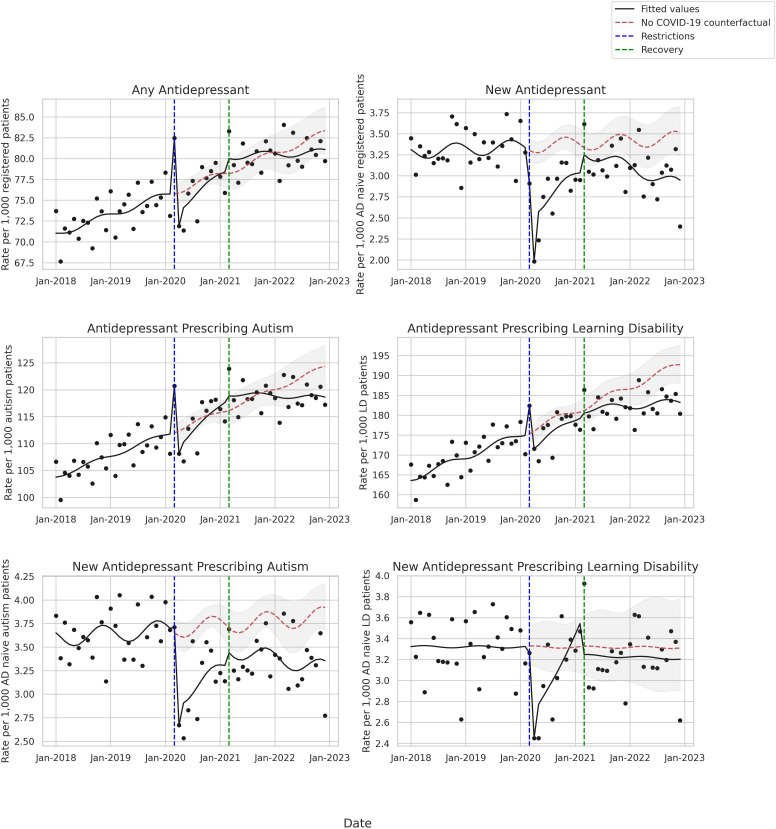
Rate of antidepressant prescribing per 1000 among those registered patients in the general population (top) with autism (left) or with learning disability (right) from January 2018 to December 2022 adjusted for long-term seasonality and trend. On top, the left column is the whole population and the right column is those newly started on an antidepressant without an antidepressant prescription for the previous 2 years. The vertical dotted lines represent the restrictions period (March 2020–February 2021) and the recovery period (March 2021–December 2022). The dotted red line is the predicted (no COVID-19) counterfactual. AD, antidepressant; LD, learning disability.

From January 2018 to February 2020, antidepressant prescribing was increasing in the general population at a relative rate of 0.3% (95% CI 0.2% to 0.3%) per month (figure 1, table 2) (an increase in model fitted rate from 71.0 to 82.5 per 1000).

**Table 2:** Relative change in antidepressant prescribing during the restrictions period (March 2020–February 2021) and recovery period (March 2021–December 2022) adjusted for seasonality and long-term trend

	Pre-COVID-19 monthly slope(95% CI)	Restrictions period		Recovery period	
		Level shift (95% CI)	Change in slope (95% CI)	Level shift (95% CI)	Change in slope(95% CI)
All prescribing	0.3% (0.2% to 0.3%)	−3.4% (−7.4% to 0.6%)	0.3% (−0.2% to 0.9%)	2.3% (−1.8% to 6.5%)	−0.5% (−1.0% to −0.1%)
Autism prescribing	0.3% (0.2% to 0.4%)	−3.4% (−7.1% to 0.3%)	0.4% (−0.1% to 0.9%)	1.2% (−2.8% to 5.3%)	−0.7% (−1.2% to −0.3%)
LD prescribing	0.3% (0.2% to 0.3%)	−2.9% (−5.4% to −0.4%)	0.2% (−0.1% to 0.5%)	0.8% (−1.8% to 3.4%)	−0.4% (−0.7% to −0.1%)
New prescribing	0.1% (−0.1% to 0.3%)	−24.8% (−33.5% to −15.1%)	1.5% (0.0% to 3.0%)	9.1% (−0.3% to 19.4%)	−2.2% (−3.7% to −0.7%)
Autism new prescribing	0.1% (−0.0% to 0.3%)	−21.7% (−32.8% to −8.9%)	1.0% (−0.8% to 2.8%)	5.8% (−3.9% to 16.5%)	−1.4% (−3.2% to 0.5%)
LD new prescribing	−0.0% (−0.3% to 0.3%)	−23.3% (−32.7% to −12.4%)	2.8% (1.3% to 4.3%)	−8.4% (−18.5% to 3.0%)	−2.7% (−4.2% to −1.2%)

LD, learning disability.

Allowing March and April to be outliers, there was no significant change in prescribing rates of antidepressants during the restrictions. Comparing restrictions to recovery, there was a slope change of −0.5% (95% CI −1.0% to −0.1%), but in March 2021 (recovery start) and in December 2022 (study end) there was no evidence of a difference in rate between our model and the rate assuming no pandemic had occurred (online supplemental figure S6). Looking at the postrestriction average RR, we did not find evidence that the COVID-19 pandemic was associated with a change in antidepressant prescribing trend in the general population (RR 1.00 (95% CI 0.97 to 1.02)) (figure 1, online supplemental figure S1).

### Trends in new prescribing

Prior to restrictions, the rate of new prescribing was stable (figure 1, table 2). There was a decrease in new prescribing (a −24.8% (95% CI −33.5% to −15.1%) step change) at the beginning of restrictions but this returned to within the expected range by March 2021. Comparing restrictions to recovery, there was a −2.2% (95% CI −3.7% to −0.7%) slope change.

In the last month of the study period there was −0.6 (95% CI −0.9 to −0.2) per 1000 decrease in new antidepressant prescribing compared with the rate assuming no pandemic (online supplemental figure S6). The average RR of new prescribing post restrictions was 13% below expected if COVID-19 had not happened (RR 0.87 (95% CI 0.83 to 0.93)) (figure 1, online supplemental figure S1).

### Autism and learning disability subgroups

#### Autism overall prescribing

In those with a record of autism, findings were similar to the overall population. Antidepressant prescribing was increasing (0.3% (95% CI 0.2% to 0.4%)) per month (from 103.8 to 120.8 per 1000 from January 2018 to February 2020) (figure 1, table 2). The average over the postrestriction period was not significantly different from the no COVID-19 counterfactual (RR 0.99 (95% CI 0.97 to 1.01)), but in December 2022 the rate was −5.7 (95% CI −9.7 to −1.7) per 1000 below expected without the pandemic (online supplemental figure S6).

#### Autism new prescribing

In those with autism, new prescribing was stable pre-COVID-19 and 12% lower than expected had pre-COVID trends continued (RR 0.88 (95% CI 0.83 to 0.92)) (online supplemental figure S2). At the start of the recovery, there was no difference between the model-based fitted value with the pandemic and pre-COVID-19 trends, but in December 2022, the difference was −0.6 (95% CI −0.9 to −0.2) per 1000 (online supplemental figure S6).

#### Learning disability overall prescribing

In those with a record of learning disability, the baseline rate of any antidepressant prescribing was also increasing (0.3% (95% CI 0.2% to 0.3%)) from 163.6 to 182.4 per 1000 from January 2018 to February 2020 (figure 1, table 2). The average over the postrestriction period was not significantly different from the no COVID-19 counterfactual (RR 0.98 (95% CI 0.96 to 1.00)). In December 2022, however, the model fitted value with the pandemic rate was −9.5 (95% CI −14.6 to −4.4) per 1000 (online supplemental figure S6) below that which was expected had COVID not occurred.

#### Learning disability new prescribing

Unlike in the general population or those with autism, there was a significant increase in slope during restrictions (2.8% (95% CI 1.3% to 4.3%)) among those with learning disability, suggesting an increase in prescribing in this population over this period. In December 2022, there was no evidence of any difference from expected trends nor overall evidence that new prescribing post restrictions was lower than pre-COVID-19 trend (RR 0.96 (95% CI 0.87 to 1.05)) (online supplemental figure S2).

### Demographic and clinical subgroups

**Figure 2:**
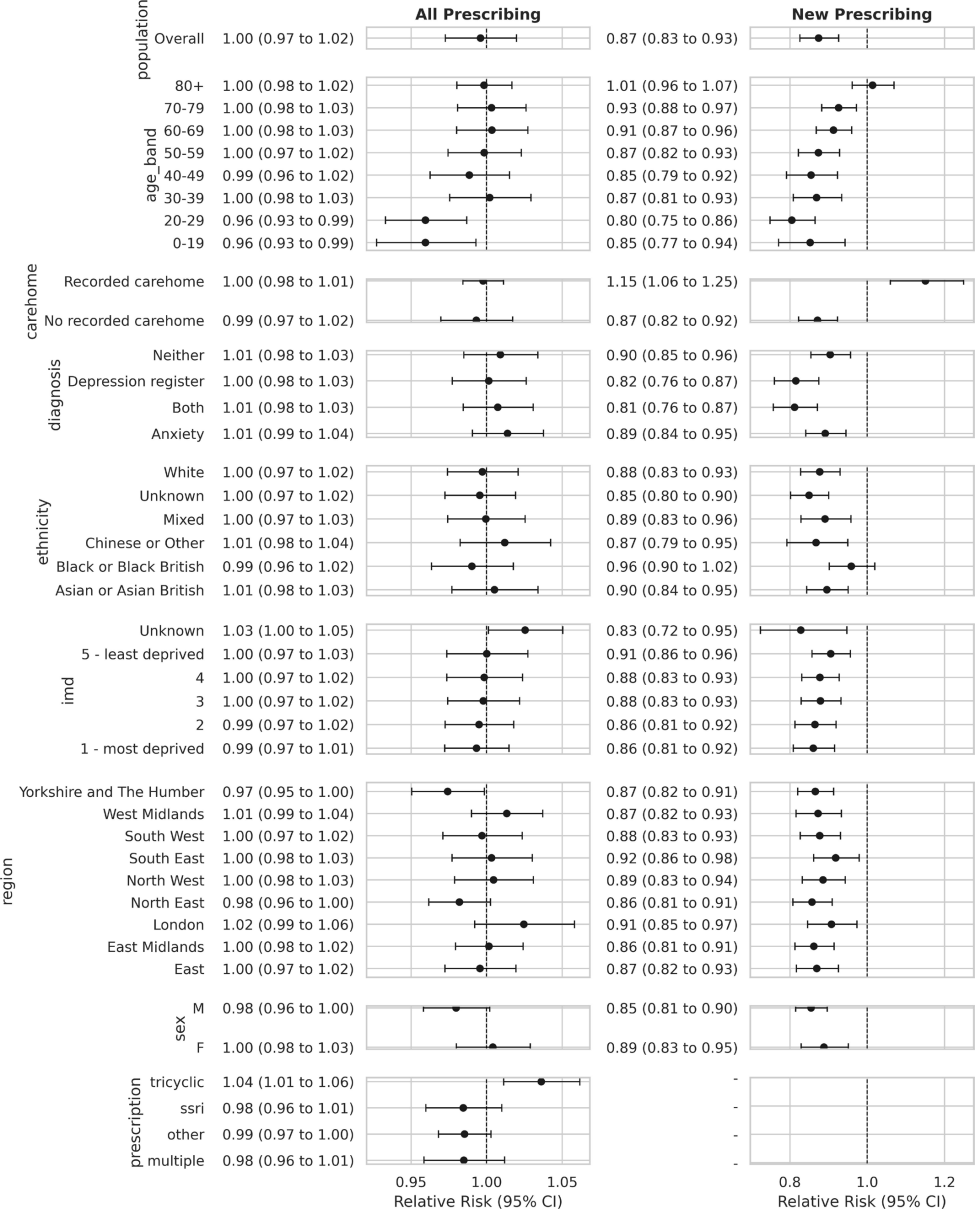
Overall and new antidepressant prescribing relative risks from March 2020 through December 2022 compared with the predicted (no COVID-19) counterfactual, by demographic subgroup. A relative risk of 1 represents no change from pre-COVID-19 trends. IMD, Index of Multiple Deprivation.

Although antidepressant prescribing in the general population post restrictions was in line with pre-COVID-19 trends, it was 4% lower for under 30s compared with the no COVID-19 counterfactual (0–19 and 20–29, both RR 0.96 (95% CI 0.93 to 0.99)) (figure 2 and online supplemental figure S4).

New antidepressant prescribing in the general population was 13% lower than pre-COVID-19 trends would have predicted, and the difference with the counterfactual trend generally increased with decreasing age (80+ RR 1.01 (95% CI 0.95 to 1.07) vs 20–29 RR 0.80 (95% CI 0.75 to 0.86)) (figure 2).

For those in a care home there was a 15% increase in new prescribing (figure 2). In the final month of the study period, this meant a difference of 1.8 (95% CI 0.7 to 2.9) per 1000 antidepressant-naive patients (online supplemental figure S7).

There was no evidence that COVID-19 was associated with a difference from pre-COVID-19 trends in antidepressant prescribing for any of the diagnosis groups (depression, anxiety, both, neither) (online supplemental figure S3).

### Sensitivity analysis

Data from OpenPrescribing showed an increase in the rate of overall antidepressant prescribing from 96 to 117 per 1000 from September 2018 to December 2022 (OpenPrescribing data were available starting in September 2018). Our ITSA analysis of these data (online supplemental figure S5) also showed that dispensed prescribing trends were in line with what would have been expected had COVID-19 not happened.

## Discussion

### Statement of principal findings

Prior to the COVID-19 pandemic, antidepressant prescribing was increasing about 0.3% per month. We found that the pandemic did not significantly affect this trend; however, since March 2021 (ie, recovery period) there has been a notable decrease in antidepressant prescribing overall. When we analysed specific subgroups, we found a 15% increase in antidepressant initiation (new prescribing) in patients residing in care homes. We also found that prescriptions to those aged 0–19 and 20–29 years were lower than pre-COVID-19 trends. Prescribing to patients under 18 years is often shared with secondary care services.

### Findings in context

A number of studies globally used ITSA to assess the impact of COVID-19 on mental health. Campitelli *et al* found a 1.43% increase in individuals dispensed antidepressants in nursing homes in Canada.^19^ In Israel, Frangou *et al* found COVID-19 was associated with an increase in antidepressant prescription fills.^16^ Wolfschlag *et al* found no change in antidepressant prescribing in one Swedish region although they note that Sweden did not experience a true ‘lockdown’, highlighting that the impact of COVID-19 restrictions may have been country specific.^20^

Using the UK’s Clinical Practice Research Datalink (CPRD) dataset, Mansfield *et al* found substantial reduction in depression, anxiety and self-harm primary care contacts in March 2020 that had not recovered by July 2020.^21^ We also saw antidepressant and new antidepressant prescribing were below expected trends in July 2020, but allowing more follow-up time, we found both had recovered by March 2021.

Also using CPRD, Taxiarchi *et al* found that prescribing for depression and anxiety decreased at the beginning of the restrictions period, yet they found this had recovered by the beginning of the recovery period.^22^ Carr *et al* performed an ITSA of mental illness and self-harm, including antidepressant prescribing in England,^23^ and found a 36.4% decrease in first antidepressant prescriptions in April 2020 that had recovered by September 2020. We saw a level shift of −29.8% for new prescriptions with restrictions, but we did not see new prescribing recover until later. Our ‘new prescribing’ outcome, however, includes those who had a previous prescription ≥2 years ago. This might contribute to our higher baseline rate of new prescriptions (3.3 vs 2.2 per 1000).

Macdonald *et al* used OpenSAFELY to assess antipsychotic prescribing to those in care homes, and with learning disability or autism. Comparing Q1 2019 to Q4 2021, they found decreased antipsychotic prescribing to those with learning disability or autism, and an increase in new prescriptions to those in care homes,^24^ in accordance with our findings for antidepressants.

Since 2019, NHS England has maintained indicators to monitor antidepressant prescribing to those with learning disability and we can roughly validate our numbers against theirs. Branford and Shankar^25^ found 10.3% annual prevalence (calculated as number of patients with a prescription in the last 6 months) of antidepressant prescribing in the general population and 20.7% in those with learning disability for NHS financial year 2020–2021. When we calculated prevalence in the last 6 months of NHS financial year 2021 with our data, we found prevalence rates of 12.2% and 22.8%, respectively. Small differences between analyses are normal and expected due to differences in underlying populations.

### Strengths and limitations

A key strength of this paper is its scale; using the OpenSAFELY platform we have been able to access raw, pseudonymised, single-event-level clinical events for >24 million patients in England, who are registered at NHS GP practices, that use TPP software. This allowed us to explore medication usage, diagnostic events and salient clinical and demographic information, including ethnicity, age and scores of deprivation.

There are, however, limitations to note. ITSA is a strong quasiexperimental study design that can help address confounding by using each population as its own control. But this uncontrolled ITSA cannot address competing risks. Greater awareness around inappropriate prescribing, for example, National Institute for Health and Care Excellence guidance that antidepressants should not be offered as first-line treatment for mild depression, is a competing factor that could have impacted prescribing at the same time as COVID-19. A future-controlled ITSA comparing those with severe versus non-severe depression could help contextualise this effect. Pragmatically, we assumed antidepressant prescriptions would be issued on a 4-week interval. If some patients are non-compliant or have longer prescribing intervals, their medications may not be issued every month. If the level of non-compliance changed over the course of our study or doctors changed their repeat prescribing practice, this could have impacted our results.

This research relies on accurate recording of diagnosis and clinical events within primary care records, but this is a limitation of all large Electronic Health Record database projects. Finally, this study describes rates of antidepressant prescribing, which may vary slightly from rates of dispensing or usage. However, within our sensitivity analysis, we have compared our findings with rates of antidepressant dispensing (OpenPrescribing data) and found the overall prescribing result to be similar.

### Policy implications and interpretation

In 2021, a national review by the chief pharmaceutical officer estimated that up to 10% of all medicines prescribed within the UK are no longer needed or not appropriate for continued use.^26^ This review highlighted a systemic problem of medicines issued without a documented indication. Our findings concur with this review, as we saw that up to one-third of patients issued an antidepressant do not have a diagnosis of depression or anxiety recorded within their notes, and this problem is even more prevalent in those with a diagnosis of learning disability. It is possible that GPs may have recorded other indications or recorded indications in free text.

In 2016, the STOMP initiative was launched whose^4^ aim was to reduce overprescribing of psychotropic medicines within the learning disability and autism populations. While it is positive to see that the pandemic did not trigger a large increase in antidepressant prescribing in those with learning disability or autism, we saw a significant increase in new antidepressant prescribing in patients who live in care homes, a particularly vulnerable group that includes those with learning disability. We need to continue to focus on these vulnerable populations to ensure that antidepressants are used appropriately.

We have shown we can use the OpenSAFELY platform as a tool to monitor the impact of directives at a comprehensive level within ‘at-risk’ patient populations across the UK. With appropriate permissions and where appropriate support can be obtained from relevant professional bodies, the OpenSAFELY platform is also technically capable of providing audit and feedback information about clinical practice, and changes in clinical practice, at single sites to support improvements in patient care.

## Information governance

NHS England is the data controller of the NHS England OpenSAFELY COVID-19 service; TPP is the data processor; all study authors using OpenSAFELY have the approval of NHS England.^27^ This implementation of OpenSAFELY is hosted within the TPP environment which is accredited to the ISO 27001 information security standard and is NHS IG Toolkit compliant.^28^

Patient data have been pseudonymised for analysis and linkage using industry standard cryptographic hashing techniques; all pseudonymised datasets transmitted for linkage onto OpenSAFELY are encrypted; access to the NHS England OpenSAFELY COVID-19 service is via a virtual private network connection; the researchers hold contracts with NHS England and only access the platform to initiate database queries and statistical models; all database activity is logged; only aggregate statistical outputs leave the platform environment following best practice for anonymisation of results such as statistical disclosure control for low cell counts.

The service adheres to the obligations of the UK General Data Protection Regulation and the Data Protection Act 2018. The service previously operated under notices initially issued in February 2020 by the Secretary of State under Regulation 3(4) of the Health Service (Control of Patient Information) Regulations 2002, which required organisations to process confidential patient information for COVID-19 purposes; this set aside the requirement for patient consent.^29^ As of 1 July 2023, the Secretary of State has requested that NHS England continue to operate the Service under the COVID-19 Directions 2020.^30^ In some cases of data sharing, the common law duty of confidence is met using, for example, patient consent or support from the Health Research Authority Confidentiality Advisory Group.

Taken together, these provide the legal bases to link patient datasets using the service. GP practices, which provide access to the primary care data, are required to share relevant health information to support the public health response to the pandemic and have been informed of how the service operates.

## Supplementary material

10.1136/bmjment-2024-301378 online supplemental file 1

## Data Availability

Data may be obtained from a third party and are not publicly available.
